# The Evaluation Scale: Exploring Decisions About Societal Impact in Peer Review Panels

**DOI:** 10.1007/s11024-016-9290-0

**Published:** 2016-02-09

**Authors:** Gemma E. Derrick, Gabrielle N. Samuel

**Affiliations:** Educational Research, Lancaster University, Lancaster, LA1 4YL UK

**Keywords:** Societal impact, Peer review, Evaluation frameworks, Impact, Qualitative

## Abstract

Realising the societal gains from publicly funded health and medical research requires a model for a reflexive evaluation precedent for the societal impact of research. This research explores UK Research Excellence Framework evaluators’ values and opinions and assessing societal impact, prior to the assessment taking place. Specifically, we discuss the characteristics of two different impact assessment extremes – the “quality-focused” evaluation and “societal impact-focused” evaluation. We show the wide range of evaluator views about impact, and that these views could be conceptually reflected in a range of different positions along a conceptual evaluation scale. We describe the characteristics of these extremes in detail, and discuss the different beliefs evaluators had which could influence where they positioned themselves along the scale. These decisions, we argue, when considered together, form a dominant definition of societal impact that influences the direction of its evaluation by the panel.

## Introduction

The 2006 Cooksey review of publicly funded health research stated that the UK was at risk of “*failing to reap the full economic, health and social benefits of public investment in health research*” due to health research not being adequately translated into health outcomes (Cooksey 2006). Similar calls have been made in other countries where the economic benefit of investing in health and medical research have been realised by government policymakers and academics alike (Donovan 2008). One such strategy to increase the societal returns from publicly funded research is to include a formal assessment of research returns in peer review evaluation processes and link the outcomes to the allocation of funding. As such, there are currently moves by public funding bodies to evaluate research in terms of both scientific and societal impact (Smith 2001) as part of research’s social contract with society (Gibbons et al. 1994; Nowotny et al. 2001; Wolfendale 1993). However, without a strong precedent for formal, reflexive (Dahler-Larsen 2012), ex-post evaluation of societal impact, questions remain about how evaluators would navigate the peer review process of these outcomes in terms of resolving their values about what constitutes excellence in the societal returns from research.

Whereas a number of conceptual models and theories have been proposed to understand the process of impact realisation and, in turn, used to guide its evaluation, actual empirical investigations of the assessment of societal impact, where the results are linked to funding outcomes, are rare (Bornmann 2012, 2013; Holbrook and Hrotic 2013). Problems associated with access to peer-review panel deliberations, and a lack of formal frameworks incorporating criteria of the societal impact of research with which to investigate, have made conducting this type of empirical research difficult. However, the UK’s 2014 Research Excellence Framework (REF2014) for the first time included a formal assessment of societal impact. Therefore, by using this framework, this article provides one of the first empirical investigations of how evaluators expected to weigh the main concerns expressed in the literature about the evaluation of the societal impact of research, when faced with the task of evaluating this criterion formally.

In particular, this study describes five separate decisions expressed by evaluators prior to the evaluation process taking place that must be resolved within peer review group discussions about formally assessing the societal impact of research. This article links hypothesised issues about impact evaluation, which have already been discussed widely in the literature (Bornmann 2013), with broader concerns about how evaluator definitions (Huutoniemi 2012; Langfeldt 2006), biases (Langfeldt 2004) and behavioural tendencies (Langfeldt 2001) contribute to group-based peer review processes. In the absence of a firm, reflexive precedent for evaluation, questions are raised regarding the reliability of review outcomes and the interplay of evaluator viewpoints. It is important to explore these questions prior to the assessment process so as to gain an insight into the baseline values evaluators hold regarding societal impact evaluation, and so to understand the nature of these tensions independent to the development of an evaluation culture that evaluators quickly acquire during evaluation panel discussions (Olbrecht et al. 2007; Langfeldt 2001). This is also important as it is this committee culture that ultimately influences the review outcomes (Kerr et al. 1996; Langfeldt 2004), and the future evaluation behaviours of peer reviewers in similar situations that will require them to use their experience of evaluating the societal impact of research from this situation. In addition, by describing the range of tensions that exist prior to the formal evaluation process taking place, this research provides an insight into what tensions will dominate the group discussions within the review process, and hypothesise about how these may be resolved by the panel.

Although the results presented here may provide a guide to interpret the REF2014 impact evaluation results, this is not its primary goal. Instead, the results discussed in this article aim to use the process of impact assessment as a way of understanding the range of tensions faced by evaluators regarding the assessment of societal impact, in the absence of prior experience or methods of benchmarking this measure.

In the next section, we discuss the relevant literature regarding the process of peer review and, in particular, the concerns regarding the evaluation of societal impact, as opposed to the more traditional, scientific impact of publicly funded research. In the absence of prior studies (Bornmann 2012, 2013; Holbrook and Hrotic 2013), we discuss research of panel-based peer review processes that will be used to interpret our results. The methods section will describe the approach employed for this study. In particular, it will describe the REF2014’s formal, ex-post “impact” criterion. The results section will discuss the analysis of 62 semi-structured interviews with REF2014 evaluators for health, medical and biomedical research submissions, and prior to the formal evaluation process taking place. This section will also introduce the conceptual model employed to discuss the differing tensions anticipated by evaluators that emerged from the interview analysis. In the final section we discuss how the five decisions evaluators make about societal impact of research prior to the assessment process taking place, are represented on the conceptual evaluation scale. Further, we explore how the conceptual model can be employed to understand group-, panel-based, peer review assessment of the societal impact of research.

## Literature Review

### *Panel-Based Peer Review and Societal Impact*

Panel peer review reaches a common judgement through what Olbrecht and Bornmann (2010) described as mutual social exchange, where the final judgement is based on the common judgement of all evaluators. Academic peer review is regarded as a “system(s) of institutionalized vigilance” (Merton 1973) in the self-regulation of the research community. Academic legitimacy of review outcomes is achieved by including experts (or peers) on the panel; a perception that the evaluation process is focused on the cognitive content of submissions and is independent of reviewers’ social identities (Langfeldt 2004), theoretical biases (Huutoniemi 2012) and tolerance for risk (Lee 2012), and that the standards of research excellence adopted by the peer review panel are fair. As long as these perceptions of impartiality are maintained, the results of the review outcomes are legitimised and accepted by the research community (Tyler 2006). However, as the definition of research excellence evolves, to one that includes interpretations of societal impact, this legitimisation of peer review outcomes is threatened as panel evaluators are required to employ untested, new evaluative criteria to make assessments.

The incorporation of “societal impact” can be described as a Kuhnian revolution for research evaluation criteria (Luukkonen 2012). As such, in order to achieve a revolutionary change towards including considerations of societal impact, the idea must be constantly debated, re-defined and reformed before the new paradigm is adopted. An important implication of using peer review when evaluating societal impact is, therefore, that during a period of time in which paradigm shift is occurring, there are multiple scientific contenders who support highly variable viewpoints, making it challenging to achieve consensus within peer review committees (Luukkonen 2012). Additionally, when assessing societal impact, evaluators are required to assess the value of the wider impact of the research, using a different perspective, one as a public stakeholder, rather than as a peer. Therefore, without a clear precedent for effective evaluation, differences in what is believed to constitute a societal impact are likely to be more pronounced within a discipline where there are already conflicting viewpoints about what constitutes excellent research, such as health (Derrick et al. 2011; Haynes et al. 2011).

It is even more unclear how research evaluators, when faced with an untested, and unknown evaluation criteria such as “societal impact”, will approach an evaluation process in such a way that will be viewed as impartial and legitimate by the wider research community. Past research has shown that peer review panels regard any new evaluation criteria as separate from concepts of “excellent research” (Huutoniemi 2012), through studies of panel assessments of the concept of “frontier research” (Luukkonen 2012). Luukkonen (2012) also suggested that in panel assessment situations, submissions proposing unorthodox claims of “frontier research” that were contrary to the personal experience of the panel reviewers, needed to meet a higher burden of proof during assessment. Indeed, Lamont (2009) argues that the cognitive value of submissions cannot be assessed in a way that is separate from the assessor’s “sense of self and relative positioning”, suggesting that the interpretation of excellence in societal impact may be heavily based on an evaluator’s personal experiences, and values (Lamont 2009). Reviewer bias, where evaluators do not interpret or apply evaluative criteria in identical ways, may be more pronounced in situations where personal conceptions of the criteria are deemed as more reliable yardsticks for evaluation than untested, unfamiliar guidelines (Lee 2012; Olbrecht and Bornmann 2010; Lamont and Huutoniemi 2011; Huutoniemi 2012). Given the ubiquitous nature of how research influences societal outcomes (Smith 2001; Ovseiko et al. 2012), it is still unclear how this exaggerates any biases in peer review processes when considering these new knowledge evaluation criteria. In addition, in situations where experiences, definitions and values of societal impact vary between individual panellists it is difficult to predict how these differences would be resolved during group evaluation discussions.

### *Issues with Evaluating Societal Impact*

Despite moves towards a wider definition of “research excellence” that incorporates aspects of both scientific and societal impact, there has been a general reluctance (Holbrook and Hrotic 2013) to integrate the formal assessment of the societal impact of research into panel-based evaluation practices. Instead, evaluation systems still prioritise measures of scientific impact as critics doubt the possibility of sensitively, objectively and accurately evaluating the societal impact of research (Nolan et al. 2008), as research can influence society differently between fields (Derrick et al. 2011), indirectly (Haynes et al. 2011) and over varying time periods (Smith 2001).

A number of problems with successful societal impact evaluation have been hypothesised and identified through the development of various models of societal impact realisation and evaluation, such as the Payback (Buxton and Hanney 2008), and SIAMPI models (Spaapen and van Drooge 2011), of which it is not necessary to discuss in detail here. However, common difficulties and decisions for societal impact evaluators which are essential to consider in this paper include issues of causality, attribution and knowledge creep.

Issues of causality and attribution relate to the understanding that societal impact is rarely realised in a linear, organised fashion but instead through the complex interplay of serendipity, luck, and complex networks (of researchers and non-researchers) interacting on various knowledge translation levels (Nutley et al. 2007; Weiss 1979). Specifically, causality refers to the difficulty in attributing impacts to a specific cause (Martin 2007); whereas the attribution problem means that it is not clear what proportion of impact should be attributed to different research, organisations, or researchers (Bornmann 2013). Finally, knowledge creep refers to the risk associated with assessing only short term impacts and overlooking the variety of ways the same research may have a future impact.

In terms of evaluation, therefore, it is difficult to navigate from which specific piece of research, or researcher-interaction, the societal impact originated, and therefore who to reward as having realised the higher level of excellence (i.e. who did what, and how much is it worth). Previous research suggests that overall impact can be mapped into major and minor contributions (Penfield et al. 2014), implying a hierarchical value to some inputs than others and failing to consider the possibility that impact may not have been realised without some minor contributions. This would therefore suggest that minor contributions be similarly valued to major ones, thus highlighting the difficulty in evaluating these different aspects under a formal, societal impact assessment criterion.

At the point of assessment, the extent that impact has progressed will influence how evaluators would assess it as significant. Penfield et al. (2014) use the example of a discovery of a new drug to highlight the issue of evaluating pathways to impact, where following the discovery of a new drug, an amount of pre-clinical work is required, followed by clinical trials in different phases (Phase 1, 2 and 3), before regulatory approval can be gained and the drug offered on the market (Penfield et al. 2014). Once the drug is adopted by health professionals and applied to patients, then impacts on the health and wellbeing of individuals, populations, and nations (through increased GDP or savings/costs to the health system) can be realised. However, the time at which the impact assessment takes place, either before or after the Phase 3 clinical trials, or after the drug has been offered to patients, influences the value placed by evaluation panels on the societal impact criteria. Questions remain as to whether the final health benefits of such a drug are valued more than the discovery of the drug and/or successful clinical trials. Evaluators’ personal experiences of this impact progression, as well as the perspective in which they are considering the impact (as an individual, organisation or nation) will also influence the value that is placed on each stage of the development of this impact. This is especially pronounced for health research, where the realisation of societal impact frequently requires actors from other fields (Niederkrotenthaler et al. 2011), can be reflected in a number of quantitative and qualitative indicators (Ovseiko et al. 2012; Dembe et al. 2013), and involve both academic and non-academic actors (Penfield et al. 2014).

## Methods

### *The UK REF2014 and the Impact Evaluation Process*

The REF2014 provided a basis for resource allocation, accountability for public investment in research, and benchmarking information for the higher education sector (HEFCE 2011). Three main assessment components include 65% of the total score dedicated to a traditionally driven peer review of research “Outputs”; and 15% to an assessment of the Higher Education Institute’s (HEI) “Environment”. The third component, 20% to an assessment of “impact”, formed the basis for this investigation. The REF2014 defined “impact” as “*…an effect on, change or benefit to the economy, society, culture, public policy or services, health, the environment or quality of life, beyond academia*” (HEFCE 2011). The assessment process was governed by one of the 4 overarching Panels. In this study, we used Main Panel A and its subpanels to investigate the evaluation of impact (societal impact).

### *Impact Case Study Assessment Guidelines*

Assessment of the “Impact” criterion was conducted according to the generic definition given above, by reviewing 4-page case studies submitted by each university, as well as an impact template that described the wider university strategy of facilitating the translation of research into impact. The structure of the 4-page case studies was tightly controlled by a template supplied by HEFCE, where universities must nominate pieces of underpinning research and then proceed to explain how this research has had an impact. This underpinning research must be considered to have reached a threshold of no less than 2 stars in quality (“*quality that is recognised internationally in terms of originality, significance and rigour*”).

To guide the panel’s assessment of impact, evaluators have been asked to make an overall judgement of impact against 2 criteria; significance and reach. Significance is defined as the “*intensity of the influence or effect*”; whereas “reach” is described as “*the spread or breadth of influence or effect on relevant constituencies*”. The criterion of “reach” is not restricted to purely geographical terms, nor in the number or context of particular beneficiaries, but instead on the spread or breadth to which the potential constituencies have been affected. The assessment of impact is awarded either 1 of 5 star profiles, where the lowest rating (Unclassified, 0 stars) is where “*…the impact has little to no reach or significance, or was ineligible, or not underpinned by excellent research produced by the significant unit*”, and the highest (4 stars) is where the impact “*…is outstanding in terms of its reach or significance*”.

### *Recruitment*

The Higher Education Funding Council for England (HEFCE) was informed and supportive of the research project. This coordination meant that all interviewees felt comfortable and adequately informed as to the aim and objectives of the research project.

A total of 215 individual evaluators from across Main Panel A were identified (www.ref.ac.uk) and invited to participate. Main Panel A included 6 subpanels related to health and medical research, these were: (1) Clinical Medicine; (2) Public Health, Health Services and Primary Care; (3) Allied Health Professions, Dentistry, Nursing and Pharmacy; (4) Psychology, Psychiatry and Neuroscience; (5) Biological Sciences; and (6) Agriculture, Veterinary and Food Sciences. Each evaluation panel included a number of international and UK-based experts, as well as traditional academic evaluators, and research user (stakeholder) evaluators. The user evaluators were predominantly from outside the academic sector and represented a variety of private, public or charitable sectors that either use university-generated research, or commission or collaborate with university-based researchers.

Invitations were originally sent via email, resulting in a total of 62 evaluators agreeing to participate in the interviews (28.8% response rate). All interviewees were provided with a participant information sheet and written consent was obtained prior to commencement of the interviews. Ethics approval was granted on 22 November 2014 from the Brunel University Research Ethics Committee (2014/4), prior to the interviews taking place.

### *Interviews*

Interviews were performed prior to the evaluation of the impact and were conducted via telephone, skype, or face-to-face, and were recorded, and transcribed for analysis. Interviews lasted between one and two hours, were semi-structured, and were conducted by GD during January until March 2014.

The interviews were designed to explore how participants viewed, valued and engaged with the concept of research impact, and its evaluation. In order to do this, interviews incorporated a number of themes. Each theme comprised one, main, overarching question, followed by a series of ‘prompts’. Interview themes were based around common issues currently discussed in the academic literature about the evaluation of research impact and peer review. These included: interviewees’ personal definition of impact; implicit bias in research evaluation (Langfeldt 2004); influence of evaluation guidelines (Langfeldt 2001); productive interactions as indicators of impact (Spaapen and van Drooge 2011); intentions and strategies for assessing impact and overcoming difficulties (including causality, attribution and time lag issues) (Buxton and Hanney 2008); anticipated difficulties and power relationships (Lamont and Huutoniemi 2011); the role of different types and levels of impacts (Bornmann 2013); and indicators of impact, attribution and causality (Buxton and Hanney 2008). Interview questions also drew on the participants’ previous research and peer-review research evaluation experience, and the influence of research impact in these situations. In the interests of confidentiality, all participant information was coded and then entered into NVivo for analysis.

### *Analysis*

Analysis of interview data was approached using inductive reasoning employing the inductive approach of grounded theory (Charmaz 2006; Strauss 1987). The analysis (or coding) of data was based on two inter-linked rounds: overview analysis and detailed analysis (Strauss 1987). Overview analysis consisted of memo-making and broad coding. Extensive memo-making was employed by the interviewer directly after each interview. Broad coding by both GD and GS proceeded by scanning the interview transcripts for relevant ideas and themes. Discussion between the two authors suggested no major disagreements. Codes were compared with emergent themes from the memo-making, and three over-arching themes were developed. These were: value (the value, or types of values, evaluators place on research impact); process (how evaluators view the research and impact process); and evaluation (evaluators’ views related to how impact will be assessed in REF2014, and the issues they envisage). Themes were then used to inform detailed coding of the full transcripts during the second round of coding. Detailed, line-by-line analysis of the interview transcripts was employed using NVivo software. Coding was carried out via constant comparison, which was continual, rigorous and allowed for developing and refining of conceptual categories as theory was developed. Duplicate coding by both GD and GS was cross-checked to ensure reliability of data. Where possible, n values are included in the discussion below, only when a separate analysis node was available within the qualitative codebook for quantification of the issue being discussed.

## Results

### *Evaluation Without Precedence*

The evaluators showed a variety of values, views and beliefs about societal impact (“*I’m still not convinced everybody shares exactly the same definition of what constitutes impact or where they place the weight of it or if it’s impact or isn’t*” P3Imp1), including a strand of uncertainty which was often expressed explicitly; “*I’m very happy to describe the quality of the research [but] the valuing of impact is something I have no idea about*” P0P2OutImp1. The newness of the criteria highlighted that the assessment of societal impact or the “*impact stuff*” made evaluators “*nervous*” as distinct from the more traditional modes of research assessment were “*what we cut our teeth on*”.

And I don’t believe that we know how to do it – you have to contrast this with the assessment of outputs which is really just reviewing, which is bread and butter stuff for an academic. That’s what we cut our teeth on, that’s what we do every day…..Whereas this impact stuff, we just don’t know. So I feel a little bit nervous about it (P0P2OutImp1).

Many evaluators spoke openly about the concerns that they had regarding evaluating the societal impact (n=26). Evaluators spoke either explicitly about this unease (“*Whereas this impact stuff, we just don’t know. So I feel a little bit nervous about it*” P0P2OutImp1), or more implicitly in terms of their uncertainty surrounding how to approach the evaluation process (“*we are all a bit working in the unknown at the moment*” P3OutImp5); (n=16). Alongside this, evaluators also spoke about their apprehension about the newness of the criteria (n=17) (“*this is a completely new exercise..[..]..we actually haven’t got a clue what we’re going to do; we have never done this before*” P3OutImp1), and their relative inexperience as societal impact evaluators (“*for a lot of us it’s not within our experience directly*” P5OutImp4). This was not to say that participants had not in the past evaluated societal impact, however, they commented that in these cases, societal impact was not considered a core assessment criteria but as a “tick box”.

The research council introduced this criteria, it’s just a tick box….So they got this box, you may just tick it [and] we tell them why this [research] will have amazing impact on humanity for the rest of eternity, and everybody ticks that… (P2OutImp5).

For other interviewees, this meant that the consideration of societal impact was simply disregarded as an unimportant “dead weight”;

But that sometimes becomes such a dead weight around the necks of the people making the decisions that it outweighs everything else…. (P2Imp1).

In these cases, the strategy for evaluation was primarily associated with assessing the “feasibility” of societal impact, rather than the outcome itself; “*I think we’ll be, what we will do in terms of assessment, is to kind of make a judgement to what extent it is a realistic impact from the underlying study that people present*” (P2OutImp2). This required that participants base their decisions on information provided about the applicant’s track record, and the potential interest of any results to academia, both of which are already used more generally to assess research excellence, allowing participants to evaluate proposals using their traditional tools of research assessment. In contrast, for ex-post evaluation such as under the REF2014 there was a widely held assumption that evaluating the societal impact was “*….an interesting experiment and I’m quite concerned about how we are actually going to evaluate it*” (P2OutImp6), and that as “*…new territory for all of us, and none of us know – we are going to learn on the job, I think*” (P4OutImp6).

The range of beliefs and values about specific aspects of societal impact realisation, and how these will contrast each other during the evaluation, are explored in more detail below using the proposed conceptual model of impact evaluation, the evaluation scale.

### *The Evaluation Scale*

To aid the discussion of the contrasts expressed by interviewees about the assessment of societal impact, the analogy of an “Evaluation Scale” is used. This scale supports two, extreme ends that prior to the evaluation discussions taking place are held in equilibrium, before evaluation discussions taking place, (1) the Quality-focused evaluation; and (2) the Societal impact-focused evaluation. In a balanced state, prior to the evaluation process, the evaluation scale represents the societal impact evaluation process, in the absence of any precedent for societal impact evaluation, and the absence of a firm committee culture that usually develops during the evaluation process (Olbrecht et al. 2007). The scale will become unbalanced during the evaluation discussions, depending on evaluator arguments relating to a number of decisions identified during the interviews. These decisions include: The importance of the underpinning research in evaluating societal impact, the value of the impact versus the “right impact”, whether impact is linear, controllable or serendipitous, the role of push factors, and whether impact is measurable or unmeasurable. Once all these decisions have been made by the peer review panel, the scale will be adjusted between the two evaluator extremes and will then represent the dominant definition of societal impact formed by the peer review panel. This dominant definition, we will argue, influences the direction of the societal impact evaluation, and may provide a lens with which to interpret evaluation outcomes.

Below, we first describe the two extremes of the evaluation scale, and then move on to outline the range of tendencies that evaluators described in relation to a number of key decisions to be resolved during the evaluation discussions.

### *The Quality-Focused and Societal Impact-Focused Evaluation*

For the quality-focused evaluation, the quality of academic research was a necessary underpinning component of societal impact assessment. On this extreme, for societal impact to occur, “high quality” academic research was considered essential or the “*sine qua non*”: “*I think research will only have an impact if it’s of high quality. I think quality is the ‘sine qua non’ of impact*” (P0OutImp2). Here, societal impact was viewed as intrinsic to the definition of research “quality”. The tendency to view societal impact in this way refers to current modes of evaluation of research excellence, which embed the criteria of impact as “*one of the dimensions of quality*” (P0P2 OutImp1), along with other attributes such as originality, rigour and significance of the work to advance knowledge. As societal impact was already considered as part of their definition of research excellence, this mode showed no need to employ new evaluation approaches to assessing the ex-post impact, tending instead to be preoccupied with using as assessment of the quality of the underpinning research as a proxy for societal impact evaluation.

The use of the underpinning research as a proxy emphasised a belief of this evaluation scale extreme, that scientific impact was a necessary precursor to excellent societal impact. Here, this belief system was vehemently expressed by interviewees, and justifications concentrated on the belief that only excellent research contributed to believable societal impact: “*I think that certainly the quality of the research is an important part. It’s a critical part. You have to have the highest quality research in order for it to be believable and repeatable*” (P0OutImp5). The evaluation approach for this mode thus involved finding a balance between the assessment of societal impact and an appreciation of the quality of the research that underpinned it.

On the contrary, societal impact-focused evaluation was not preoccupied with the scientific impact of the underpinning research: “*the quality of the research has [no] role at all in ensuring the impact of the research*” (P6OutImp2). Indeed, evaluators towards this extreme felt that it is “*not true*” that “*low quality research cannot be impactful*” (P1Imp1). Instead, scientific and societal impact were considered separate, ‘independent’ entities and it was ‘important to discriminate’ between them: “*I think what’s important really is to discriminate between research and impact*” (P0OutImp6).

The separation between scientific and societal impact was characteristic of this extreme, as was the belief that each should have independent assessment criteria: “*route to impact is going to be on grounds that are entirely independent of the criteria that we will be using to judge the quality of the papers that led to it*” (P2OutImp3). This meant that, unlike quality-focused evaluation, societal impact-focused evaluators were unlikely to be “*swayed by the underlying science*”. Indeed, case studies that were seen to be “*trumpeting the science*” were considered distracting from the societal impact assessment:

Some [case studies submitted from their institution] have made the mistake of telling us what the original research was rather than what happened next. Where did it go? Trumpeting the science paper from 1998 rather than telling us what happened for health or wealth as a result of it (P1OutImp3).

Therefore, unlike the quality-focused mode, evaluators tending towards the societal-impact-focused mode were unlikely to consider criteria typically associated with traditional evaluation approaches (e.g. rigour, methodology and originality (Luukkonen 2012)) when assessing societal impact.

What may be a product or an end result of research has different criteria associated with it because what you’re looking for here is a societal change…whereas the research…it’s quite different..[it]..is all around rigor and methodology and the quality of the idea and making sure that the methods and the quality of the idea match (P0OutImp6).

Instead, in this mode, assessment was dependent on the extent that the societal impact could be reliably attributed to the underpinning research (causality); and their interpretations of the concepts of “significance” and “reach” supplied by HEFCE. However, applying consistent definitions of significance and reach by all evaluators were also anticipated as an issue for societal impact evaluation:

At this stage of the process in some ways I think we should have almost a binary scoring system: “did this have impact in your view or did it not? And if it did, it did, so what’s the problem?” Because actually the significance and the reach aspects are the ones that are going to give it a four, three or two star (P6OutImp2).

In the interviews, the evaluators rarely adopted all the views characterised by either the quality- or the societal impact-focused evaluation mode. Rather, they positioned themselves somewhere between these two extremes, adopting positions along the scale in relation to a number of decisions that we describe below.

### *Decision 1: The Importance of the Underpinning Research in Evaluating Impact*

For more quality-focused evaluators, the importance of underpinning research when evaluating impact was driven by an underlying value system depicting a strong link between scientific and societal impact. Indeed, 29 participants explicitly expressed that there was a relationship between the quality of the research, and its societal impact. An additional 17 participants felt that a relationship exists, but that it would be “*weak*”. The strength by which some evaluators held onto this belief system was highlighted by the use of words such as ‘*should*’ and ‘*I would hope*’ when expressing their considerations of the relationship between scientific and societal impact: “*excellent impact should depend on excellent research*” P1OutImp6; and “*you would hope they were synonymous wouldn’t you*” (P3OutImp5). Of the participants who explicitly declared a relationship between scientific and its resulting societal impact (n=29), 3 of these expressed a “*hope*” that this relationship would be maintained. In the more extreme cases, this belief reflected an underlying moral assumption that societally impactful (sic) research extends only from research that is of excellent quality when evaluated traditionally, whereas ‘hope’ related to a strong desire for it to be the case.

This value system, in turn, reflected evaluators’ beliefs about how societal impact should be valued. Thus, the basis of this value system was that quality has intrinsic worth or ‘*importance*’ over and above impact: “*I can see some problems in areas…where it could be a bit doubtful whether this kind of emphasis on impact might actually distort what is really important*” (P0P1OutImp1). Indeed, in many cases, evaluators displayed a pride in their own research that could be inextricably linked to this quality. This personal, somewhat anecdotal experience only served to reinforce their associated value system towards research quality:

Interviewer: Can you describe for me what type of or what part of your research or research career you are most proud of?

Evaluator: I’ve had two papers published in *Nature* and two in *Science* (P6OutImp1).

The tendency to value the quality of the underpinning research was paramount when assessing societal impact because they could not “*take impact seriously*” unless it “*meets the minimum standards of quality*” (P2OutImp3). This was because research excellence represented the pinnacle of the reason why research is conducted. Without this pinnacle there is nothing to value or assess because “*it [the research] doesn’t actually tell you anything, so why would it have an impact*” P4Imp1. Indeed, without quality, evaluators spoke about the impact as being ‘hollow’ (“*if you don’t have the strong research base then the impact seems to be rather hollow*” (P2OutImp8)); ‘potentially dangerous’ (“*potentially dangerous actually, especially because if these experimental results are weak or uncertain…then any impact they have would be positively a negative thing*” (P4OutImp5)); ‘negative’ (“*I think that poor quality research can only have negative impacts*” (P4OutImp5)); and/or ‘not believable’ (“*you have to have the highest quality research in order for it to be believable*” (P0OutImp5)).

Whilst many evaluators considered the quality of the underpinning research as necessary for societal impact, views were more varied when it came to whether all quality research led to societal impact. For some evaluators, excellent research would result in an impact: “…*good quality research usually does impact*” (P3OutImp10), although this could occur in the future: “…*my feeling is that eventually it will but it might take a long time*” (P3Imp2). This, however, was certainly not always the case as “*…quality and impact go hand-in-hand, but not all high-quality research will necessarily have an impact*” (P3OutImp1). In fact, many evaluators with different value sets united over this, albeit for different reasons. Some evaluators related the correlation to the intrinsic nature of the research, for example, P2OutImp4 believed that “*…some excellent research will be pretty early in the research chain*” and P2OutImp6 believed that, “*some excellent research is not designed to have an impact*”.

The importance evaluators placed on the underpinning research when evaluating societal impact also had implications for the societal-impact evaluation extreme of the evaluation scale, where impact was considered separate from the underpinning research. In this way, the importance of the underpinning research in the assessment of societal impact was only such to ensure that it met the minimum two star threshold as specified by HEFCE (2011), “…*and one of the reasonable challenges is turning research into impact. Assuming that it is good enough to meet sort of minimum standards of quality that means it should be taken seriously*” (P2OutImp3). In this way, the intentions towards the assessment of societal impact were primarily concerned with other decisions that are discussed below.

### *Decision 2: The Value of the Impact Versus the Value of the “Right” Impact*

For some evaluators, the necessity for research of a high quality to underpin societal impact was guided by the assumption that impact referred to ‘*good impact*’, as opposed to ‘*negative*’ societal impact. Many evaluators (n=14) expressed concern about the potential danger of rewarding ‘*negative*’ impact. Though interviewee’s views about the definition of ‘good’ societal impact were both diverse and complex, overall, ‘good’ impacts were defined as being the desirable or the right type of societal impact.

Categorising societal impact as ‘good’ and ‘bad’ influenced how evaluators characterised the scientific-societal impact relationship. For some more quality-focused evaluators, the relationship between scientific and societal impact was not tied to quality as an essential component of societal impact such that without quality there is no societal impact. Rather, whilst ‘good’ impact was tied to quality - since “*for good impact it has to be high quality research*” (P1OutImp2), without quality, societal impact could still be realised, just “*not in the right way*” (P3OutImp4), as “…*if you want a negative impact than any old research will do*” (P1OutImp2). One of the more prominent examples used by interviewees to illustrate this was the Measles, Mumps and Rubella Vaccine debate (MMR), and many drew upon this example to demonstrate how ‘*poor science*’ can lead to a ‘*huge impact*’:

The example…is the MMR story that was poor science. It’s had huge impact, negative impact. It’s resulted in lots of morbidity amongst children plus women – people didn’t get their children vaccinated. The quality of the science was poor, but it had a huge impact in a negative way (P2OutImp9).

Alongside these views, other evaluators tending towards a quality-focus drew on the MMR story highlighted above, not to point towards a ‘good’-‘bad’ societal impact distinction, but more as a cautionary note to highlight a much looser correlation between scientific and societal impact. For these evaluators, MMR was a prime example of an instance when bad quality led to ‘big’ societal impact; destabilising the strong belief that scientific and societal impact was synonymous:

You could even argue that really low-quality research like the stuff that came out on MMR a few years ago, which has been completely discredited from an academic perspective, has had a really massive impact (P3Imp2).

At the other evaluation scale extreme, was the tendency by the societal impact-focused mode to see all impacts as societal impact. Indeed, assuming that the impact case study met the minimum two star research quality threshold, and that its causality and attribution was sufficiently supported by the evidence supplied, then societal impact was considered absolute. In these situations, interviewees stated that they wouldn’t know what was going to prevent them from giving all case studies four stars (a rank of outstanding) automatically: “*I don’t envisage everything being four stars. I just think we shouldn’t be shy about giving the higher scores*” (P1OutImp5).

This decision on the scale distinguished between the value of societal impact, where only positive societal impacts were rewarded with high evaluation (quality-focused evaluation), and awarding all societal impacts with high evaluations (societal impact-focused evaluation) regardless of the value, or effect, that that impact had on society.

### *Decision 3: Impact as Linear, Controllable or Serendipitous*

A major underpinning factor influencing evaluators’ opinions was related to whether to view impact as related to ‘outside factors’ separate to the research, or something that was viewed rationally, therefore related to the quality of the research.

Towards the quality-focused extreme, evaluators envisaged a ‘pipeline’ from high quality research to societal impact – “*a sort of translational pipeline is the okay term that tends to get used for taking a scientific discovery and pushing it towards some sort of laboratory test, new drug, or whatever, which, I guess, many people would view as some sort of impact*”(P1OutImp5). Thus, the relationship between scientific and societal impact hinged upon the idea that “*impact requires that you generate the evidence and then that you, in turn you get into guidelines and the people start using that information to change their practice*” (P4Out1). This idea relates to a knowledge-driven model of health policymaking (Weiss 1979) which presumes that knowledge is used in a rational way based on the quality of the research, and that because knowledge exists, it will be used. Achieving societal impact from research was thus ‘*straightforward*’ and, to a degree, uncomplicated, and therefore so would be its evaluation.

Research was done, showed the benefits of [the evidence], got into the clinical guidelines, and over time you can track the proportion of the relevant professionals who are implementing the better evidence. It’s quite straightforward in fact (P3OutImp8).

Indeed, in some instances, evaluators perceived societal impact as “*almost immediate - as soon as something is published and seen to be useful, you will find [this institution] using it within months probably*” (P1OutImp7).

Other evaluators who tended towards a societal impact extreme, spoke about external factors playing a role in its realisation, for example, P3OutImp6 commented that “…*impact depends to some extent…upon other wider factors within society*”. The decision for evaluators here therefore surrounded the extent that these chance occurrences should influence the societal impact evaluation. In this way, evaluators who tended towards the societal impact-focused extreme did not assume that societal impact consists of a series of rational decisions on which scientific research findings can be brought to bear (Greenhalgh and Wieringa 2011). Instead, evaluators recognised the ‘messiness’ of the societal impact process, and that impact was dependent on a whole range of ‘uncontrollable’, ‘outside factors’ (or “*forces*”) that needed to be overcome: “*there are forces out there that try to inhibit development as well as encourage it*” (P0OutImp6).

In terms of aiding societal impact realisation, some evaluators acknowledged the influence of ‘serendipity’ (n=18). This was the concept that, in contrast to the quality of the research, an element of ‘*luck*’ or fortunate happenstance materialised in impact realisation and was evident once the research was completed: “…*it can often be that coffee you have with somebody at the right moment and the information passing that way*” (P1Imp1). For other evaluators, the uncontrollable nature of societal impact realisation was less related to the beneficial forces of serendipity, and more associated with a dependence on wider society, which created a barrier to societal impact. Such factors were linked to whether research was ‘*fashionable*’: “…*often it’s not to do with the quality of the research. It’s a whole lot of other things about kind of workplace cultures and what is the kind of fashionable thing of the time*” (P3OutImp5); or ‘*timely*’; “*…I guess what we don’t see is those good ideas that somehow weren’t timely, and which have sort of fallen by the wayside*” (P2OutImp4). They were also linked to the receptiveness of stakeholders, and interviewees were aware that stakeholders may not want “*to take the research up*” representing a barrier to societal impact: “…*you could do excellent research that wasn’t impacted because stakeholder groups didn’t want to take it up*” P4Imp1. In many cases the underlying barrier to this was commercial:

Say a pharmaceutical company does buy a product and puts it into their company and then they decide not to develop it. Well it may – they won’t be because of scientific reasons, necessarily not developing it, but maybe just for financial reasons (P0OutImp6).

Another barrier to impact realisation was the complexity of the policy process, and the political reasons why research may not achieve impact. This was, as one interviewee described, the case for the black report on health equalities which “…*got buried by the government at the time…So I suppose you’ve got to be a bit careful because people might make all their best efforts and it [research] may still not be effective*” (P3Imp1). In addition, the difficulties navigating the policy process and the influence of serendipity in ensuring societal impact were discussed more generally:

That kind of linear approach is very, very rare indeed. The way it is instead is that findings accumulate over a period of time and either the weight of evidence in the end wins the day or a moment arrives when the politicians have made up their minds that they want to go in a particular direction, they are looking around for the evidence to support their decision (P2Imp1).

The commonality among beliefs in external forces influencing societal impact realisation, was that its existence upset their belief of the rational link between scientific and societal impact (previously described). However, the consequences of this were that evaluators formed different opinions about societal impact assessment. For those where considering the uncontrollable nature of societal impact required an evaluation that reflects the role of serendipity, there was a tendency to shift towards the quality-focused end of the scale and to use traditional research quality assessment tools, since the role of chance occurrences was “…*why we still have to judge it alongside the robustness of the work and the quality of the work and those sorts of things, not just on did it change something?*” (P2OutImp3). In contrast, for evaluators tending towards the societal impact-focused extreme, fortunate happenstance played the same role as other ‘barrier’ factors to societal impact realisation, and reinforced a belief that a valuable component of assessing societal impact was considering how these factors can be overcome with the use of ‘push’ factors, or productive interactions (Spaapen and van Drooge 2011). We discuss these push factors in more detail below.

### *Decision 4: Push Factors and Assessing Impact*

Towards the quality-focused evaluator extreme, the assessment of societal impact was influenced by a belief that a researcher’s role in ensuring societal impact was limited solely to providing high quality research, whereas it was the responsibility of other, non-researchers to use this as evidence to pursue societal impact. Some evaluators (n=22), considered ‘*doing impact*’ as outside of the role of researchers, and/or separate to ‘*doing research*’. In these situations, traditional evaluation tools were deemed sufficient for assessing societal impact, as this underpinned a belief that for research to ‘*make it*’ to societal impact, research was to ‘picked up’ or ‘pulled’ into the societal impact domain independent of the researcher. This ‘pull’ relied on the right dissemination method, either in the right academic journals (“*of course publication in scientific journals. In academic journals, that’s the main way of promoting our research*” (P6OutImp1)); to the ‘right audience’ (“*so obviously if you’re talking to a medical doctor you’re going to use a different language than if you’re talking to a politician, to if you’re talking to a service user and you’ve got to kind of understand that I think*” (P3Imp1)); or in the ‘*correct language*’:

I also think there are people on the ground who will go to conferences and hear people speak, or who will read professional journals which won’t necessarily be at all what we think of as an academic paper….because those people would read those things and say, “oh, you know, maybe there is something here and I should be thinking about this, or maybe we could make changes on the basis of this (P3OutImp5).

The underlying assumption here was that if research was disseminated correctly, societal impact would happen linearly.

At the other end of the evaluation scale, was the assertion that alongside a researcher’s traditional role, research needed to be ‘pushed’ in order to produce societal impact because “…*however good the research is, if the system is not absorptive then you won’t have an impact*” (P1Imp2). Getting research acknowledged often required “…*a little effort*” (P1OutImp4), to ensure societal impact “…*does happen rather than sort of by Brownian motion*” (P1Imp1). As P1 Outimp4 stated:

Getting from the university stage of research out to the end impact has so many steps in it, not all of which are easy. They require a little effort and somebody championing them from one end or the other.

This assertion recognised that “*…you can’t just assume that it will happen through happenstance, there need to be some mechanisms in place*” (P1Imp1). The factors required to ‘push’ the research towards societal impact could take a number of forms, though most encapsulated the idea of ‘*building relationships*’ or ‘*partnerships*’ with stakeholders, including industry, government policymakers and/or patient groups. The strength of such relationships was vital for researchers to be able to “*reach out across any barrier*” (P0OutImp5). As P2Outimp4 stated:

Unfortunately in the real world things often turn on whether or not you’ve got someone’s ear in high places - whether that would be the trust chief executive or the public health agency chief executive or the permanent secretary…a lot of impact still is built around personal relationships.

In addition to partnerships were researcher activities, such as research advocacy: “*advocacy is really important…knowing how to use the ideas that you have generated to inform the policy debate*” (P2OutImp4). Interviewees understood that linking research with policy outcomes was complex, and achieving this type of societal impact required a certain amount of “*campaigning and lobbying at national and local levels for change*” (P3Imp1). In contrast to tendencies towards the quality-focused extreme, evaluators recognised that the strength of these relationships was independent of the academic reputation of: the researcher, their affiliation, or any assessment of traditional research quality (scientific impact):

Sometimes the newer universities might have much stronger links with employers or with other organisations…so I don’t think it’s always the case that the top ranking universities would always have the biggest impact (P3Imp1).

Whilst many evaluators recognised the importance of productive interactions in societal impact realisation, there was more variation in terms of whether these activities should be valued as societal impact. Whilst some evaluators dismissed interactions as evidence of societal impact alone, others felt that their contribution should be recognised as essential societal impact precursors. For these evaluators, the best way to assess impact was to reward researchers for moving the research forward and “….*levering it to the next stage*: *[whatever] gets the research being taken up and moving it forward, that has to be considered valuable. Maybe the question we should be asking is whether enough effort has gone into that in the past and levering research into its next stage*” (P0OutImp6). Considering successful productive interactions as impact tended the evaluation towards the societal impact-focused evaluation scale extreme.

### *Decision 5: Measurable Impact Outcomes Versus Unmeasurable Impact Journeys*

The final factor which influenced the evaluation scale was whether evaluators valued societal impact as a single, measureable outcome, or as a process or journey that, in many cases, is impossible to be measured. For some evaluators, societal impact was viewed as a ‘journey’ or ‘process’. In one example, an interviewee discussed this by distinguishing between a conceptualisation of societal impact as “*a verb*” rather than as “*a noun*”, where the “*noun*” concept favoured an assessment of societal impact as a measurable, end-product or outcome, and the “*verb*” as a non-measurable journey:

If you think of impact as a verb rather than a noun, I think it’s a lot easier to analyse. Impact is the relationships you build. It is the dialog that you have that makes you ask research questions that are subtly different from the ones you would have asked if you hadn’t linked with - whether it’s policymakers, whether it’s citizens, whether it’s industry at the beginning. So impact is not something that you have right at the end. Impact is a relationship and that attitude of mind that you have throughout the research process (P0OutImp4).

Such evaluators believed that societal impact was performed primarily by the researcher, rather than being attributable to the quality of the research itself (Samuel and Derrick 2015). They did not downgrade the societal impact journey because it was immeasurable, but recognised the importance of productive interactions for impact realisation: “*…if something can be measured objectively then you could use that. If it can’t be measured objectively, then it shouldn’t be downgraded purely because you can’t measure it*” (P1OutImp5). As such, these views pushed evaluator discussions towards the societal impact-focused extreme.

Other evaluators had a tendency to value societal impact as an outcome rather than a longer-term appreciation of the societal impact journey: “*I should be assessing the impact of the research and not the impact of the intermediary steps*” (P2Out1). For them, productive interactions were not seen as evidence of societal impact because they didn’t provide ‘*results*’ and their impact was therefore ‘*subject to imagination*’:

These [productive interactions] are unquantifiable, so therefore it will be difficult to assess them as impact. And again, it’s subject to imagination, you don’t know how you’ve affected anything until you see the results. So the only time that you know there is an impact is when there is a result. So, therefore, just talking to people is not an impact (P6Out2).

Rather, they believed that in terms of societal impact “*…it’s not how it’s done or who does it, it’s the actual research itself that’s important*” (P5Imp1). In particular, they valued measurable societal impact outcomes stemming directly from the research. Indeed, there was a desire to see societal impact evidenced through the provision of hard, demonstrable outcomes: “*I define it [impact] as the information that arises from research publication having a quantifiable effect outside of the grouping that’s all the original publication*” (P1Imp1). These opinions thus tended to adopt a more quality-focused position on the evaluation scale, concurrently dismissing the ‘*journey*’ as ‘*not relevant*’ in terms of societal impact assessment.

## Discussion

This study describes the range of tensions about assessing the value of the societal impact of research expressed by research evaluators prior to an evaluation process taking place. This study is unique in its description of these tensions before any ex-post societal impact evaluation process, as well as in the presentation of a conceptual model that, we argue, reflects the development of the dominant definition of societal impact that will be used during panel deliberations.

The level of inexperience with navigating the societal impact criteria allowed the study to reflect upon how five decisions about societal impact evaluation would influence the formation of a new balance of the conceptual evaluation scale during the evaluation process to be formed based on interplay of social processes between evaluators. Indeed, the concept of an evaluation scale was chosen as it allows for the existence of a complex interplay of factors and values during the evaluation process (Lamont and Huutoniemi 2011; Olbrecht and Bornmann 2010; Van Arensbergen et al. 2014; Huutoniemi 2012), and in the development of a committee culture (Olbrecht et al. 2007; Langfeldt 2001) that guides evaluation. This, in the absence of the experience or a prior precedent for societal impact evaluation, holds the concerns described by participants in balance. During the assessment process and in response to the group discussions that fuel the inevitable development of a committee culture (Olbrecht et al. 2007; Langfeldt 2001), the evaluation scale will tend towards one extreme or the other in response to evaluator decisions made about societal impact. This new balance will become the precedent for future societal impact evaluations as the evaluators apply their experience gained in this assessment process to similar evaluations in the future, i.e. the dominant definition of societal impact.

Prior to the development of this dominant definition of societal impact, the inexperience of the evaluators as expressed by the participants, will lead to the various viewpoints about societal impact expressed in this article, being played against each other during group discussions. This article has described the interplay of these viewpoints using qualitative interview data in order to demonstrate their complexity. Solely using quantitative measures of the number of evaluators, who tended towards one extreme or another as a method of predicting the evaluation criteria used for the assessment of societal impact, would disregard the importance of factors about the individual evaluators who express their preference for viewpoints along the evaluation scale. Both here, and in other studies (Lee 2012; Langfeldt 2001; Huutoniemi 2012), personal experience and perceptions of criteria were considered more reliable yardsticks for assessment than untested criteria.

The baseline views captured in this article also reveal interesting insights surrounding previously hypothesised issues with societal impact evaluation such as research causality, attribution, valuation and knowledge creep. The views expressed in this article indicate that there was a potential danger in that many important minor contributions of societal impact during the “*impact journey*” that come before a later, more-downstream impact (Penfield et al. 2014), may be undervalued. However, if there is a tendency towards the societal impact-focused evaluator, then all impacts will be awarded an outstanding rating (4 stars) independent of whether it is considered a major or minor contribution. Nonetheless, a tendency to favour major societal impact contributions, risks favouring research areas and organisations that have the capacity to make these more downstream contributions, over minor contributions without which the downstream impact may not have been realised. This bias would also neglect to acknowledge the range of serendipitous events (opportunities seized by researchers), or push factors, that are acknowledged by many evaluators as important precursors of impact, as worthy of recognition in an assessment of societal impact. In addition, whether a combination of inexperience, time restraints and personal beliefs about the relationship between scientific and societal impact, may indirectly encourage the use of evaluation proxies within the experience of the evaluators in line with a quality-focused evaluator, i.e. the use of traditional considerations of scientific impact, is unknown. However, it must also be considered that the dominant definition of societal impact used by the panel will be as much the result of the persuasiveness of the argument, and the collective personal experience of the evaluators themselves (Lee 2012; Huutoniemi 2012), as the perceived academic authority and/or level of credibility of the evaluator(s) presenting the viewpoint (Lamont 2009). This is in line with previous research into group-based peer review processes that considers how panels navigate the assessment of new criteria such as “frontier research” (Luukkonen 2012).

A major strength of this study is its timing, where the inexperience expressed by participants regarding evaluating the societal impact of research provided a unique opportunity to capture evaluator’s baseline views prior to any formal societal impact assessment process. By capturing these views expressed by evaluators pre-evaluation, this research reveals approaches to societal impact evaluation that is uncorrupted by any evaluation experience gained, or a committee culture that is developed during panel discussions, ultimately influencing evaluation outcomes (Olbrecht et al. 2007; Langfeldt 2001). However, the validity of these views and the extent that they contribute to the dominant definition of societal impact represented by the proposed conceptual evaluation scale, would benefit from further interviews conducted post evaluation. In addition, further analysis that combines post-evaluation interviews with a consideration of an evaluator’s individual authority or power within the peer review panel is needed, in order to gain an appreciation of the dominance of certain viewpoints towards impact assessment over others during peer review panel deliberations. In this study, these considerations were not taken into account, and instead the extremes (quality-focused, and societal impact-focused evaluator) were considered fluid as evaluator opinions rarely encompassed all characteristics of one typology or the other for all decisions described. This suggests that the typologies themselves may change (becoming dominant or recessive) during the evaluation process. Further research that encompasses post evaluation interviews, or observations of future evaluation processes incorporating a formal societal impact criterion would investigate such questions further.
